# Pheochromocytoma-related cardiomyopathy presenting as broken heart syndrome: Case report and literature review

**DOI:** 10.1016/j.ijscr.2018.12.003

**Published:** 2019-01-09

**Authors:** Brandon Diaz, Adel Elkbuli, John D. Ehrhardt, Mark McKenney, Dessy Boneva, Shaikh Hai

**Affiliations:** a Department of Surgery, Kendall Regional Medical Center, Miami, FL, United States; b University of South Florida, Tampa, FL, United States

**Keywords:** Pheochromocytoma, Acute coronary syndrome, Takotsubo cardiomyopathy, Catecholamine-induced heart failure, Cardiac catheterization, Surgical management

## Abstract

**Introduction:**

Pheochromocytoma are neuroendocrine tumors that arise from sympathetic chromaffin cells within the adrenal medulla. They principally secrete catecholamines, potentially causing life-threatening cardiovascular complications. A myriad of symptomatology and clinical findings are associated with pheochromocytoma, including a catecholamine-induced dilated cardiomyopathy.

**Presentation of case:**

A 50-year-old woman presented with retrosternal chest pain and underwent diagnostic evaluation for acute coronary syndrome. Cardiac catheterization demonstrated patent coronary arteries and a pattern of ventricular hypokinesis consistent with takotsubo cardiomyopathy, also known as broken heart syndrome. Further imaging with abdominal CT revealed an adrenal mass. Laboratory markers supported the clinical picture of pheochromocytoma. Right adrenalectomy was performed and our patient was symptom-free at discharge on post-operative day three.

**Discussion:**

Alpha and beta adrenergic blockade are used in a critical care setting to prevent perioperative hemodynamic instability as well as catecholamine-induced heart failure in the setting of pheochromocytoma. Patients commonly require vasopressors in the postoperative period due to the rapid reduction in circulating catecholamines following resection. Discharge planning should include recommendations for genetic counseling to screen for syndromic causes of pheochromocytoma that increase the risk for other neoplasms.

**Conclusion:**

We present a case report of a rare adrenal tumor in a middle-aged woman that manifested as acute coronary syndrome. A presumptive diagnosis of takotsubo cardiomyopathy on cardiac catheterization led to further investigation. Abdominal imaging located an adrenal mass that correlated with laboratory studies positive for high levels of catecholamines and their metabolites. The tumor was excised and the patient recovered without complications.

## Highlights

• This is a case report of a rare adrenal tumor that manifested as acute coronary syndrome.
• Adrenalectomy for pheochromocytoma presents a risk for intraoperative hemodynamic instability.
• Preoperative medical care is essential to reduce intraoperative complications.
• This case exemplifies the importance of the various symptoms that are prevalent with excessive circulating adreno-receptor agents.

## 1 Introduction

Pheochromocytomas are rare neuroendocrine tumors that arise from specialized neural crest derivatives in the adrenal medulla known as chromaffin cells, or pheochromocytes. Most cases are sporadic, although syndromic forms have been described in type II von Hippel-Lindau syndrome, multiple endocrine neoplasia types 2A and B, and in some neurofibromatosis type 1 patients. Excessive and dysregulated catecholamine secretion by pheochromocytomas are described by a classic triad of headache, diaphoresis, and tachycardia as hallmarks of sympathetic overtone. In clinical practice, fewer than five percent of pheochromocytoma presentations exhibit triad symptomatology and clinicians must rely on other signs [1].

There is a recently-growing body of literature on a minority of pheochromocytoma patients who present in overt cardiogenic shock from a catecholamine-induced cardiomyopathy [[2], [3], [4], [5], [6], [7], [8], [9]]. On cardiac catheterization and echocardiography, apical ballooning is a common feature that leads many to diagnose takotsubo syndrome, also known as broken heart syndrome, a stress-induced cardiomyopathy. Takotsubo (also spelled as tako-tsubo) is not an eponym, instead it is derived from the Japanese word for “octopus trap” as the left ventricular morphology resembles an octopus fishing pot with a round bottom and narrow neck, analogous to the cardiac apex and base respectively. The Mayo Clinic criteria for takotsubo cardiomyopathy maintain that a diagnosis cannot be made in the presence of pheochromocytoma (Table 1) [10]. They express takotsubo syndrome as an entity most commonly seen in post-menopausal women who have endured significant emotional or physical stress. Conversely, catecholamine-induced cardiomyopathy secondary to pheochromocytoma should be viewed separately as it has a neoplastic source [11].

**Table 1:** The Mayo Clinic criteria for takotsubo cardiomyopathy.

Mayo Clinic Criteria for Takotsubo Cardiomyopathy [10] (all four required for diagnosis)
Transient left ventricular hypokinesis, akinesis, or dyskinesis with or without apical involvement
Absence of obstructive coronary disease or acute atherosclerotic plaque rupture on angiography
New electrocardiographic ST segment changes (elevation or depression) or troponin elevation
Absence of pheochromocytoma and myocarditis

Striking similarities between classic takotsubo syndrome and those secondary to pheochromocytoma require a work up to rule out an adrenal source. Computed tomography (CT) is the radiographic study of choice for evaluating adrenal masses. While CT provides excellent spatial and anatomical detail, some adrenal masses require further functional imaging and laboratory data to differentiate similarly appearing masses.

Laboratory analyses for urinary catecholamine metabolites vanillylmandelic acid (VMA) and normetanephrine can be used to further support the diagnosis of pheochromocytoma. These assays are sensitive to the extent that isolated urinary VMA elevations in the absence of other signs and symptoms has led to diagnoses [12]. Other adrenal medullary lesions like sporadic adrenal medullary hyperplasia can mimic pheochromocytoma with similar symptomatology and increased 24-h urinary metanephrine [13]. While an adrenal mass with elevated catecholamine metabolites is sufficient to justify an adrenalectomy, histopathologic diagnosis remains the gold standard.

Herein, we present the case of a 50-year-old woman with characteristic findings of acute coronary syndrome who was diagnosed with takotsubo-like cardiomyopathy and ultimately found to have an underlying pheochromocytoma. This case has been reported in line with the SCARE criteria [14].

## 2 Presentation of case

A 50-year-old woman with a history of hypertension and daily tobacco smoking presented with substernal chest pain complaining of a “heavy heart.” She attributed her profound anxiety and chest pain to the recent loss of a loved one. On evaluation, she was tachycardic and hypertensive. Electrocardiogram showed ST-elevations and serum troponin was elevated at 2.84 ng/mL. The emergency department consulted cardiology and initiated treatment with beta-adrenergic antagonists, nitroglycerin, and oxygen. Working differential diagnoses included ST segment myocardial infarction, acute coronary syndrome, and typical angina.

Cardiac catheterization demonstrated patent coronary vasculature, acute decompensated heart failure, and cardiogenic shock. Interventional cardiology noted an ejection fraction of 15% and described regional ventricular hypokinesis with basal sparing, altogether consistent with takotsubo cardiomyopathy. Cardiac output was depressed at 2.7 L/min, pulmonary artery oxygen saturation was 44%, and ventricular filling pressures were severely elevated. Cardiology placed an intra-aortic balloon pump (IABP), began milrinone therapy, and moved the patient to the medical intensive care unit (MICU) for further care of cardiogenic shock. Her ejection fraction improved to >50% by the following day, prompting cardiology to remove the IABP.

**Fig. 1:**
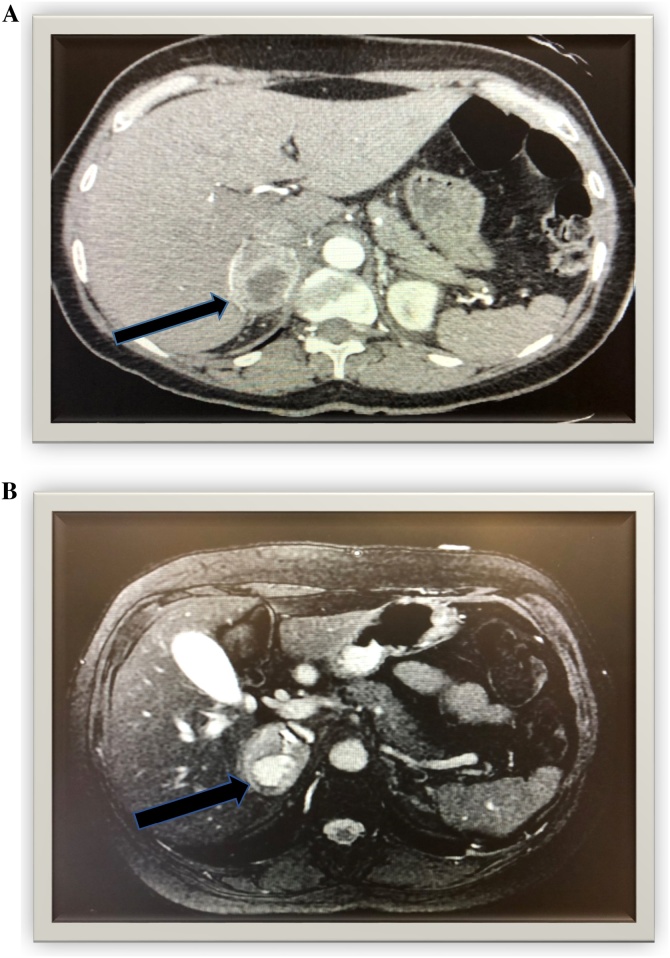
Image A: axial CT imaging with IV and PO contrast showing heterogeneously-enhancing 4 cm right adrenal mass (red arrow) with 2 cm central cystic component. Image B: axial MR imaging with IV and PO contrast of right adrenal mass (red arrow) demonstrating heterogeneous features and avid enhancement of cystic components.

Further radiographic imaging with CT and MRI revealed large right adrenal mass with central cystic components measuring 4 cm on axial scans (Fig. 1, Image A & B). Surgery evaluated the patient in conjunction with endocrinology for presumptive pheochromocytoma. Alpha-adrenergic blockade for 10–14 days before surgical intervention was begun prior to surgery to avoid adrenal and hypertensive crisis. The patient was started on selective α blockade with doxazosin therapy. She was also placed on a β-adrenergic antagonist prior to surgery. Preoperative laboratory studies for pheochromocytoma markers showed elevated urine normetanephrine, plasma epinephrine and norepinephrine, and renin activity.

**Fig. 2:**
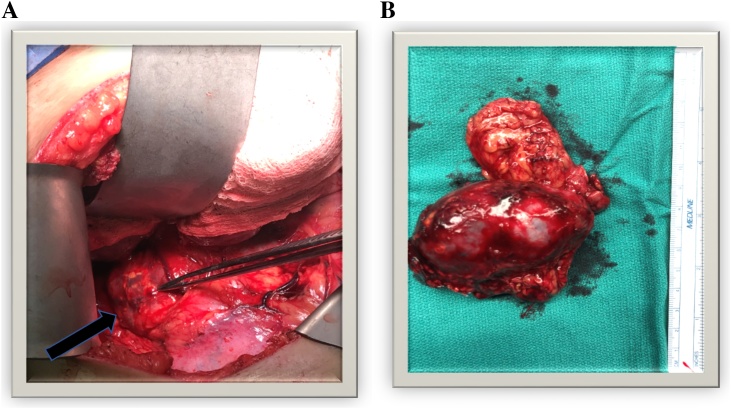
Image A: Intraoperative photo of right adrenal mass (indicated by forceps). Image B: Right adrenal mass measuring approximately 8 cm in length.

**Fig. 3:**
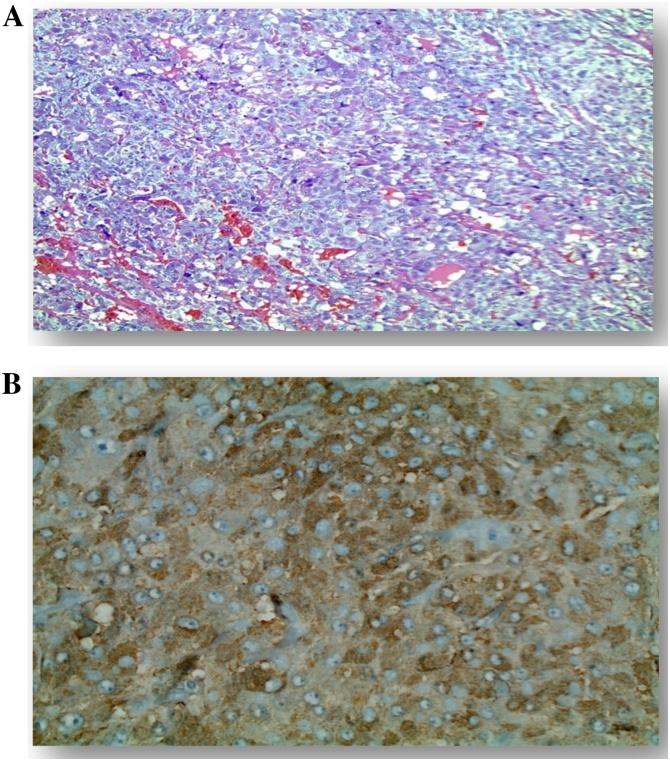
(A): Light micrograph of hematoxylin & eosin stained section (100 X H&E). Zellballen (small nests or alveolar pattern), trabecular or solid patterns of polygonal/spindle shaped cells in rich vascular network. (B): Immuno-histochemical staining with anti-chromogranin A and anti-synaptophysin antibodies. Residual adrenal gland cortex and surrounding benign fat.

After 14 days of alpha and beta blockade therapy, the patient was taken to the operating room for right adrenalectomy. A large, heterogeneous mass was visualized overlying the right kidney (Fig. 2, Image A). On removal, the specimen measured approximately 8 cm in diameter (Fig. 2, Image B). A Light microscopic examination with hematoxylin & eosin stained section (100 X H&E) showed small nests of trabecular or solid patterns of polygonal/spindle shaped cells in rich vascular network (Fig. 3, Image A). Immuno-histochemical stained section with anti-chromogranin A and anti-synaptophysin antibodies shows residual adrenal gland cortex and surrounding benign fat (Fig. 3, Image B). The patient remained hemodynamically stable throughout the operation and afterwards. She recovered without complications and was discharged from the hospital on post-operative day three with only beta blocker therapy.

## 3 Discussion

Our patient was a 50-year-old woman with a longstanding history of tobacco smoking who presented with acute coronary syndrome following the recent loss of a loved one. On cardiac catheterization, she was presumed to have takotsubo cardiomyopathy, a stress-induced “broken heart” syndrome characterized by apical ballooning of the ventricles and subsequent systolic failure. Solidifying this diagnosis requires exclusion of pheochromocytoma because a minority of patients with pheochromocytoma or paraganglioma present with a takotsubo-like cardiomyopathy [2].

Although there is no standardized protocol for the medical management of catecholamine-induced cardiomyopathy with pheochromocytoma, α and β adrenergic antagonists play crucial roles during the perioperative period. Alpha-antagonists are the historical mainstay of treatment for malignant hypertension in pheochromocytoma. Nonspecific α-antagonists such as phentolamine and phenoxybenzamine are commonly used for vasoconstrictive blockade and reduce complications from malignant hypertension to less than 3%. Specific α_1_-antagonists like doxazosin are effective as well, particularly for postoperative blockade, due to their shorter half-lives and cleaner side effect profile [15]. Aggressive α-blockade in the preoperative period can increase the risk for tumor ischemia and necrosis. Subsequent edematous changes in a necrotic pheochromocytoma can lead to interstitial hemorrhage and ultimately tumor rupture [16].

Because α-blockade primarily affects vasculature, β-blockade may be desired to improve cardiac systolic function and control tachyarrhythmia in patients with catecholamine-induced cardiomyopathy. To decrease the risk of flash pulmonary edema, cardioselective β-1 antagonists such as atenolol and metoprolol are preferred. Most importantly, α blockade should always precede β blockade to prevent hypertensive crisis from unopposed α-mediated vasoconstriction [1,[16], [17], [18], [19]].

Adrenalectomy for pheochromocytoma presents a risk for intraoperative hemodynamic instability. Patients with tumors greater than 4 cm in diameter, higher preoperative plasma norepinephrine levels, preoperative hypertension (>130/85 mmHg or MAP > 100 mmHg) with α blockade, orthostatic hypotension, and those undergoing open resection have been identified as more susceptible to complications. Postoperative vasopressor support is often necessary to maintain blood pressure in the sudden absence of excess catecholamines following resection [20,21].

Discharge planning should include recommendations for genetic counseling. Pheochromocytomas are associated with genetic syndromes MEN2A, MEN2B, von-Hippel Lindau syndrome, and Neurofibromatosis type 1, which carry with them an elevated risk of developing parathyroid hyperplasia, medullary thyroid carcinoma, and mucosal neuromas. Whether or not a genetic cause is identified, European guidelines recommend that pheochromocytoma patients be followed by endocrinology for at least ten years due to their increased risk of recurrence or new neoplastic disease [22].

## 4 Conclusion

We present a case report of a rare adrenal tumor in a middle-aged woman that manifested as acute coronary syndrome. A presumptive diagnosis of takotsubo cardiomyopathy also known as broken heart syndrome, on cardiac catheterization led to further investigation. Abdominal imaging located an adrenal mass that correlated with high levels of catecholamines and their metabolites. After preoperative preparation with α and β blockade, the pheochromocytoma was excised and the patient recovered without complications. This case demonstrates the importance of identifying atypical presentations of pheochromocytoma and safely managing perioperative complications.

## Conflicts of interest

None.

## Sources of funding

None.

## Ethical approval

Patient consent was received and ethical approval was granted by our institution’s review committee.

## Consent

Written informed consent was obtained from the patient for publication of this case report and accompanying images. A copy of the written consent is available for review by the Editor-in-Chief of this journal on request.

## Author contributions

Adel Elkbuli, Brandon Diaz, Dessy Boneva, Shaikh Hai, – Conception of study, acquisition of data, analysis and interpretation of data.

Adel Elkbuli, Dessy Boneva, John D. Ehrhardt Jr, Brandon Diaz – Drafting the article.

Dessy Boneva, Shaikh Hai, Mark McKenney – Management of case.

Adel Elkbuli, Brandon Diaz, John D. Ehrhardt Jr, Dessy Boneva, Shaikh Hai, Mark McKenney – Critical revision of article and final approval of the version to be submitted.

## Registration of research studies

This is a case report study.

## Guarantor

Dessy Boneva.

Mark McKenney.

Shaikh Hai.

## Provenance and peer review

Not commissioned, externally peer-reviewed.
